# [^68^Ga]Ga-FAPI-46 PET/CT and [^18^F]FDG PET/CT in polymyalgia rheumatica, ovarian teratoma, and reactive peritoneal lesions: a case report

**DOI:** 10.1093/bjrcr/uaag045

**Published:** 2026-10-27

**Authors:** Sofie L H Boye, Helle D Zacho, Camilla Sylvest, Bence Gabor Szilvasy, Morten Bentestuen

**Affiliations:** Department of Nuclear Medicine and Clinical Cancer Research Center, Aalborg University Hospital, 9260 Gistrup, Denmark; Department of Nuclear Medicine and Clinical Cancer Research Center, Aalborg University Hospital, 9260 Gistrup, Denmark; Department of Clinical Medicine, Aalborg University, 9260 Gistrup, Denmark; Department of Gynecology and Obstetrics, Aalborg University Hospital, 9260 Gistrup, Denmark; Department of Pathology, Aalborg University Hospital, 9260 Gistrup, Denmark; Department of Nuclear Medicine and Clinical Cancer Research Center, Aalborg University Hospital, 9260 Gistrup, Denmark; Department of Clinical Medicine, Aalborg University, 9260 Gistrup, Denmark

**Keywords:** FAPI PET/CT, FDG PET/CT, polymyalgia rheumatica, benign ovarian teratoma, peritoneal reactive changes, case report

## Abstract

In this case report, we present a 76-year-old woman who presented with an adnexal mass, ascites, and peritoneal lesions, all features highly suspicious for ovarian cancer (OC). The patient was enrolled in a clinical trial evaluating the diagnostic performance and clinical value of [^68^Ga]Ga-fibroblast activation protein inhibitor-46 ([^68^Ga]Ga-FAPI-46) PET/CT, a novel PET tracer that has demonstrated promising results in OC. The preoperative [^18^F]Fluorodeoxyglucose ([^18^F]FDG) PET/CT and [^68^Ga]Ga-FAPI-46 PET/CT showed up to moderate tracer uptake in the ovarian tumor and peritoneal lesions; however, final histopathology revealed a benign teratoma and peritoneal reactive changes. Furthermore, the patient had a history of polymyalgia rheumatica (PMR), and [^18^F]FDG PET/CT showed classic findings of PMR. [^68^Ga]Ga-FAPI-46 PET/CT revealed similar results, but with even higher tracer accumulation in affected locations. This case is the first to demonstrate the potential of [^68^Ga]Ga-FAPI-46 PET/CT in PMR.

## Introduction

Ovarian cancer (OC) is the fifth leading cause of cancer-related mortality among women in Europe, with 70%-80% of cases diagnosed at advanced stages. Imaging modalities, including ultrasound and [^18^F]Fluorodeoxyglucose ([^18^F]FDG) PET/CT, are cornerstones in the diagnosis and staging of OC. Although [^18^F]FDG PET is a valuable tool for primary staging, chemotherapy response assessment, and recurrence detection in OC, it has several limitations, including low uptake in certain OC subtypes, leading to false-negative results, and uptake in various benign conditions, resulting in false-positive findings.^1^^,^^2^ Therefore, novel PET tracers that overcome these limitations are needed to enhance the diagnostic utility of PET in OC.

[^68^Ga]Ga-fibroblast activation protein inhibitor-46 (FAPI), a novel PET tracer targeting the transmembrane protein fibroblast activation protein (FAP), has demonstrated highly promising results in the diagnostic evaluation of OC. When compared to [^18^F]FDG, FAPI PET demonstrates greater sensitivity, particularly for detecting peritoneal metastases.^2^ Fibroblast activation protein is expressed in up to 97% of OCs and is normally absent in ovarian tissue and benign conditions.^2^ However, FAPI uptake has also been observed in various benign conditions associated with inflammation and fibrosis, including rheumatoid arthritis, osteoarthritis, and vasculitis.^3^ Consequently, research on the potential application of FAPI PET in these nonmalignant conditions is rapidly evolving.^4^

## Case history

A 76-year-old woman presented with increased abdominal girth. Initial contrast-enhanced CT (ceCT) revealed an irregular adnexal mass with some calcifications, as well as ascites, bilateral pleural effusions, and possible peritoneal carcinomatosis, and OC was therefore suspected. Cancer antigen-125 (CA-125) was 1659 kU/L, and transvaginal ultrasonography demonstrated a cystic/solid ovarian tumor measuring 63 × 48 mm with increased blood flow in the solid parts. Consequently, the Risk of Malignancy Index^5^ was 14 931, and according to the International Ovarian Tumor Analysis consortium’s Assessment of Different NEoplasias in the adneXa model (ADNEX model),^6^ the risk of malignancy was 99.6%. MRI was not conducted. The patient was referred for preoperative staging with [^18^F]FDG PET/CT and was enrolled in a prospective clinical trial comparing the diagnostic performance and clinical value of [^18^F]FDG PET/CT and [^68^Ga]Ga-FAPI-46 PET/CT preoperatively in patients with a high risk of OC.^2^ Written informed consent was obtained prior to any study-related procedures. [^18^F]FDG PET/CT showed inhomogeneous up to moderate [^18^F]FDG uptake in the solid parts of the ovarian tumor (SUVmax 5.0) and low, diffuse [^18^F]FDG uptake in thickened peritoneum (SUVmax 2.0), suggesting peritoneal carcinomatosis. Similar to [^18^F]FDG PET/CT, [^68^Ga]Ga-FAPI-46 PET/CT demonstrated moderate uptake in the ovarian tumor (SUVmax 5.8) and moderate uptake in the peritoneal lesions (SUVmax 4.5) (Figure 1). The patient underwent laparoscopic salpingo-oophorectomy. Intraoperative frozen section biopsy could not rule out malignancy, as both the tumor and the cellular features were atypical. These findings were discussed with the surgeon, and it was decided to proceed with staging surgery, including hysterectomy and omentectomy. Final histopathology of surgical specimens revealed struma ovarii, a type of mature teratoma in which thyroid tissue is the predominant or sole component,^7^ and reactive changes in the peritoneal biopsy. The presentation of a benign ovarian tumor, ascites, and pleural effusion prompted the diagnosis of Meigs syndrome.^8^

**Figure 1:**
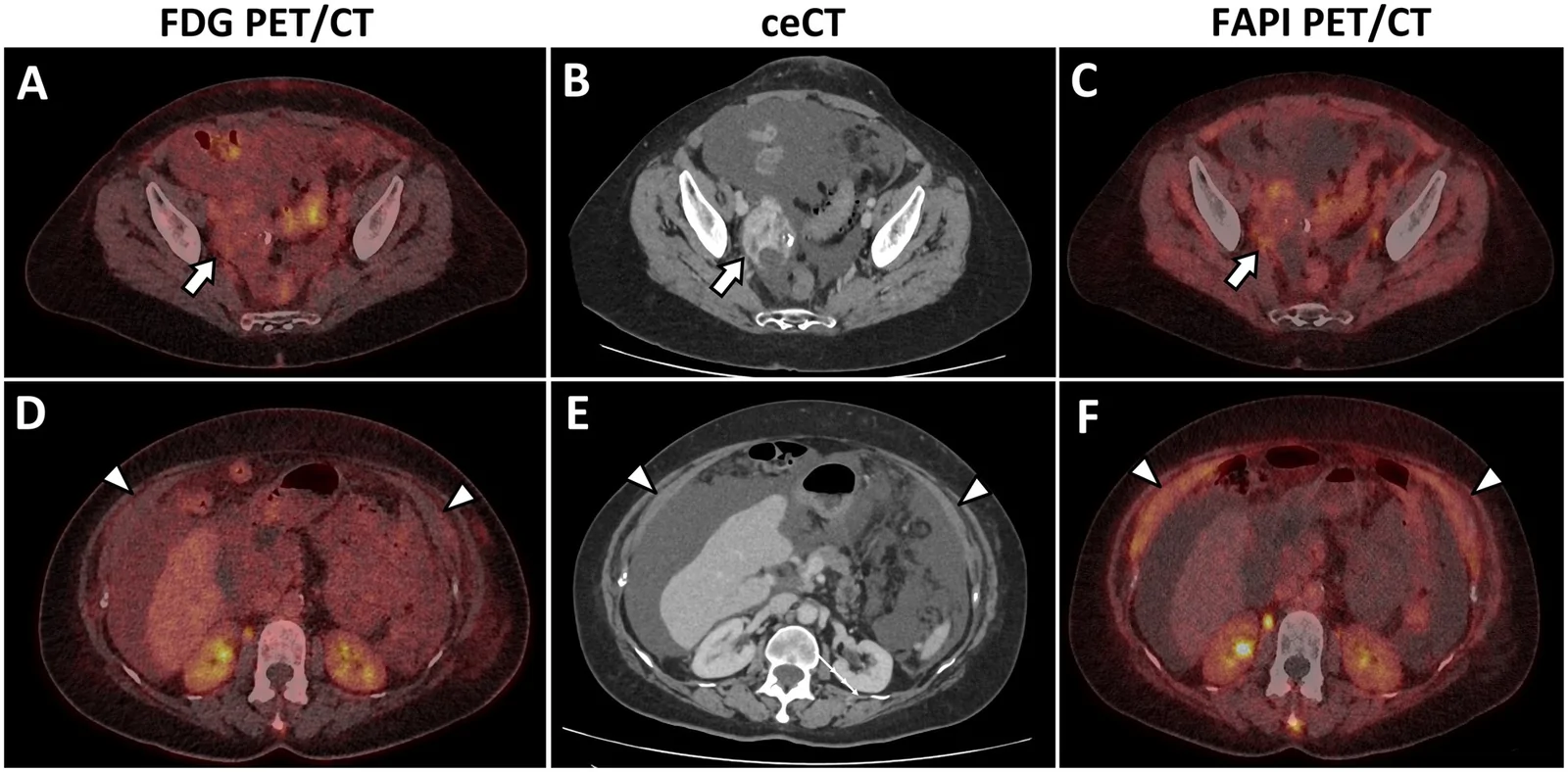
[^18^F]FDG and [^68^Ga]Ga-FAPI-46 PET/CT in benign ovarian teratoma and peritoneal thickening. (A) [^18^F]FDG PET/CT ovarian tumor (white arrow). (B) Contrast-enhanced CT ovarian tumor (white arrow). (C) [^68^Ga]Ga-FAPI-46 PET/CT ovarian tumor (white arrow). (D) [^18^F]FDG PET/CT peritoneal lesions (white arrowheads). (E) Contrast-enhanced CT peritoneal lesions (white arrowheads). (F) [^68^Ga]Ga-FAPI-46 PET/CT peritoneal lesions (white arrowheads).

In addition, the [^18^F]FDG PET/CT showed bilateral [^18^F]FDG uptake in shoulder joints (SUVmax 3.9), sternoclavicular joints (SUVmax 5.2), greater trochanters (SUVmax 3.3), ischial tuberosities (SUVmax 5.4), the pubic symphysis (SUVmax 4.9), and in cervical and lumbar intraspinal ligaments (SUVmax 4.3); findings characteristic of polymyalgia rheumatica (PMR)^9^ (Figure 2). The patient had a history of active PMR and received maintenance low-dose corticosteroid treatment (5 mg/day) at the time of PET imaging. Surprisingly, tracer accumulation in these areas was also observed on the [^68^Ga]Ga-FAPI-46 PET/CT. Moreover, the [^68^Ga]Ga-FAPI-46 PET/CT demonstrated even higher uptake values compared to the [^18^F]FDG PET/CT in the affected locations (SUVmax shoulder joints 9.8; sternoclavicular joints 12.7; greater trochanters 5.7; ischial tuberosities 10.0; pubic symphysis 8.5, and intraspinal ligaments 9.0) (Figure 2).

**Figure 2:**
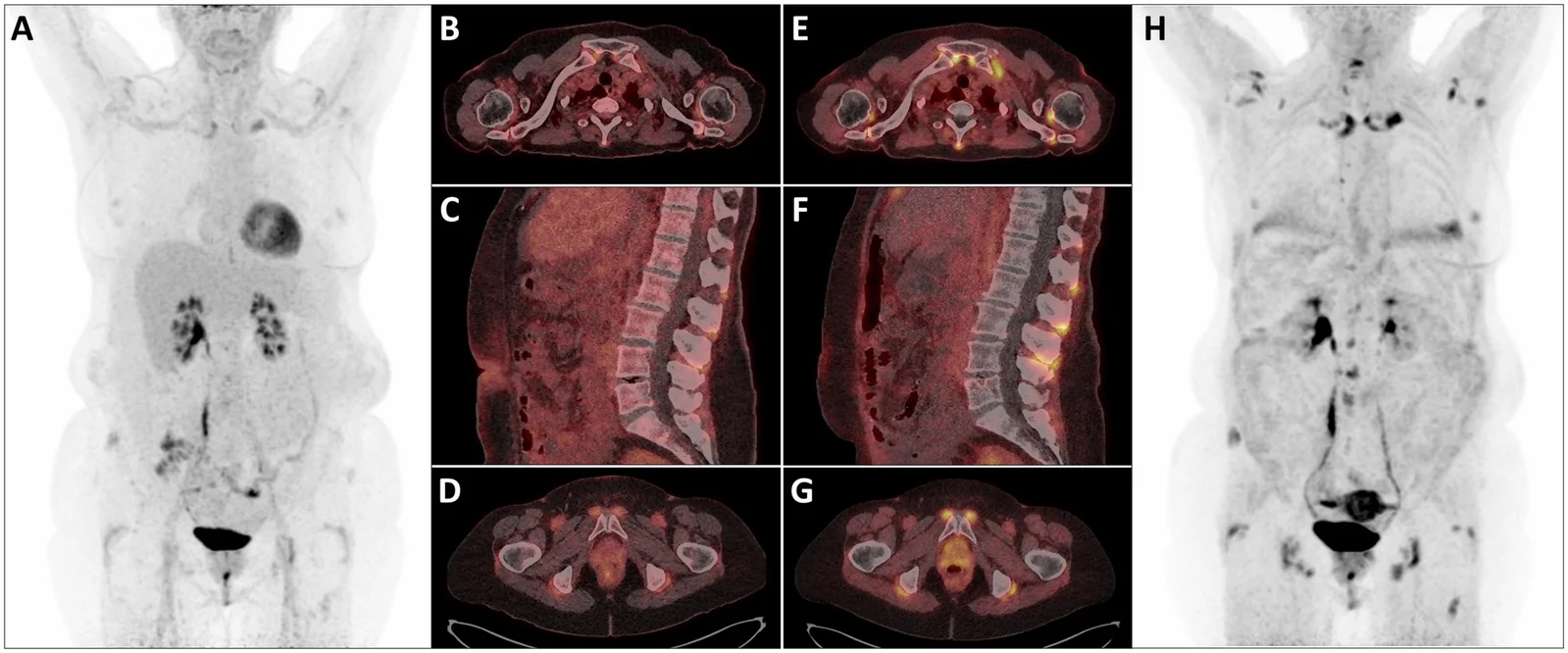
[^18^F]FDG and [^68^Ga]Ga-FAPI-46 PET in polymyalgia rheumatica. (A) [^18^F]FDG PET/CT maximum intensity projection (MIP). (B) Axial [^18^F]FDG PET/CT. (C) Sagittal [^18^F]FDG PET/CT. (D) Axial [^18^F]FDG PET/CT. (E) Axial [^68^Ga]Ga-FAPI-46 PET/CT. (F) Sagittal [^68^Ga]Ga-FAPI-46 PET/CT. (G) Axial [^68^Ga]Ga-FAPI-46 PET/CT. (H) [^68^Ga]Ga-FAPI-46 PET/CT MIP.

## Discussion

The present patient case demonstrates an example of both patient history and multiple diagnostic modalities indicating malignancy, although the final diagnosis was the benign condition, struma ovarii. Struma ovarii can mimic OC with overlapping features such as a heterogeneous ovarian mass, ascites, and elevated CA-125.^7^ To our knowledge, there is only one other reported case of benign teratoma where FAPI PET/CT was conducted, in which, unlike ours, the FAPI PET/CT demonstrated high, rather than moderate, FAPI uptake.^10^ In conclusion, malignancy must be carefully excluded in cases like the one presented, and benign conditions such as teratomas with Meigs’ syndrome should also be considered in the differential diagnosis of patients with findings suggestive of OC.

As a curious incidental finding, [^68^Ga]Ga-FAPI-46 PET/CT revealed imaging findings classically associated with the [^18^F]FDG PET/CT findings in PMR,^9^ but with markedly increased [^68^Ga]Ga-FAPI-46 uptake compared to [^18^F]FDG, with approximately 2-fold higher SUVmax values, in our case. Elevated FAPI uptake in joints has previously been described, primarily in degenerative and inflammatory conditions.^3^ However, to our knowledge, FAPI uptake has not been previously reported in cases of PMR. Although the underlying pathophysiological mechanisms for the observed [^68^Ga]Ga-FAPI-46 uptake in our case remain unclear, these observations warrant further investigation in additional patients to assess the potential value of FAPI PET in PMR.

## Learning points

- Benign conditions, such as teratomas, may present with clinical, laboratory, and imaging findings overwhelmingly suggestive of malignancy, but histopathology of lesions is always necessary to verify malignancy.
- The triad of ascites, pleural effusion, and an ovarian mass is highly suggestive of Meigs’ syndrome, a rare condition that may mimic ovarian cancer.
- [^68^Ga]Ga-FAPI-46 PET/CT may be a promising new imaging modality for evaluating PMR, but the pathophysiological mechanisms remain unclear, and further studies in additional patients are warranted.
